# Supplementary figures and images for: Outborn newborns drive birth asphyxia mortality rates—An 8 year analysis at a rural level two nursery in Uganda
Source: PLOS Glob Public Health. 2023 Nov 8;3(11):e0002261. doi: 10.1371/journal.pgph.0002261 (PMC10631647; doi:10.1371/journal.pgph.0002261)

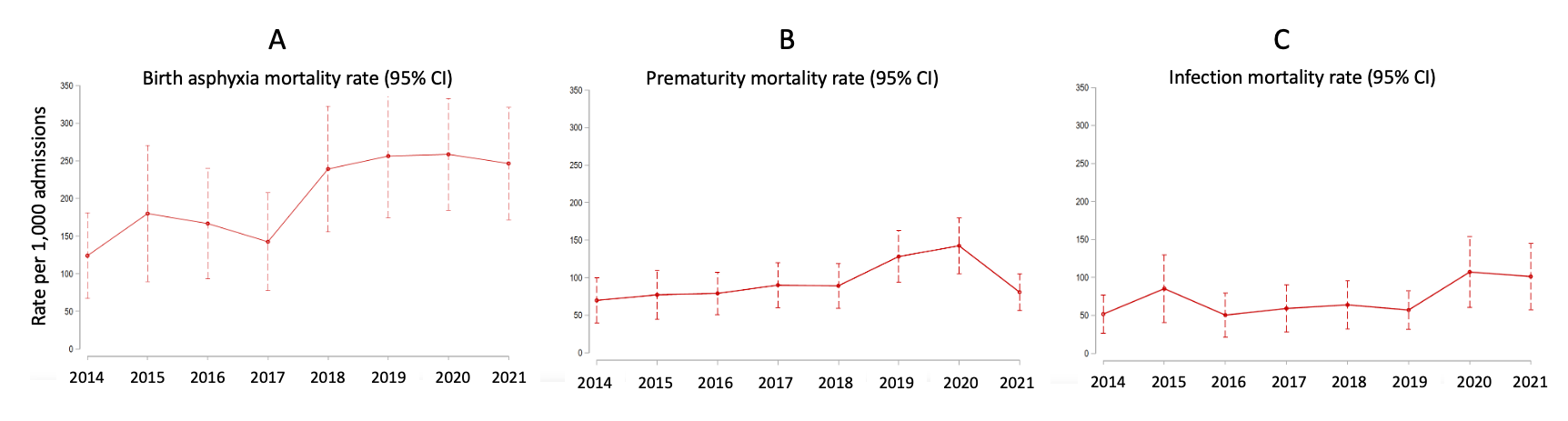

Supplement: S1 Fig — (A- birth asphyxia, B- prematurity and C- infection). Peak mortality for each diagnosis was in 2020 during the early COVID-19 pandemic. While mortality has returned to baseline for premature infants (B) and is decreasing back towards baseline for neonates with infection (C), the mortality rates for birth asphyxia patients (A) has steadily increased over time. (TIF) [file pgph.0002261.s001.tif]

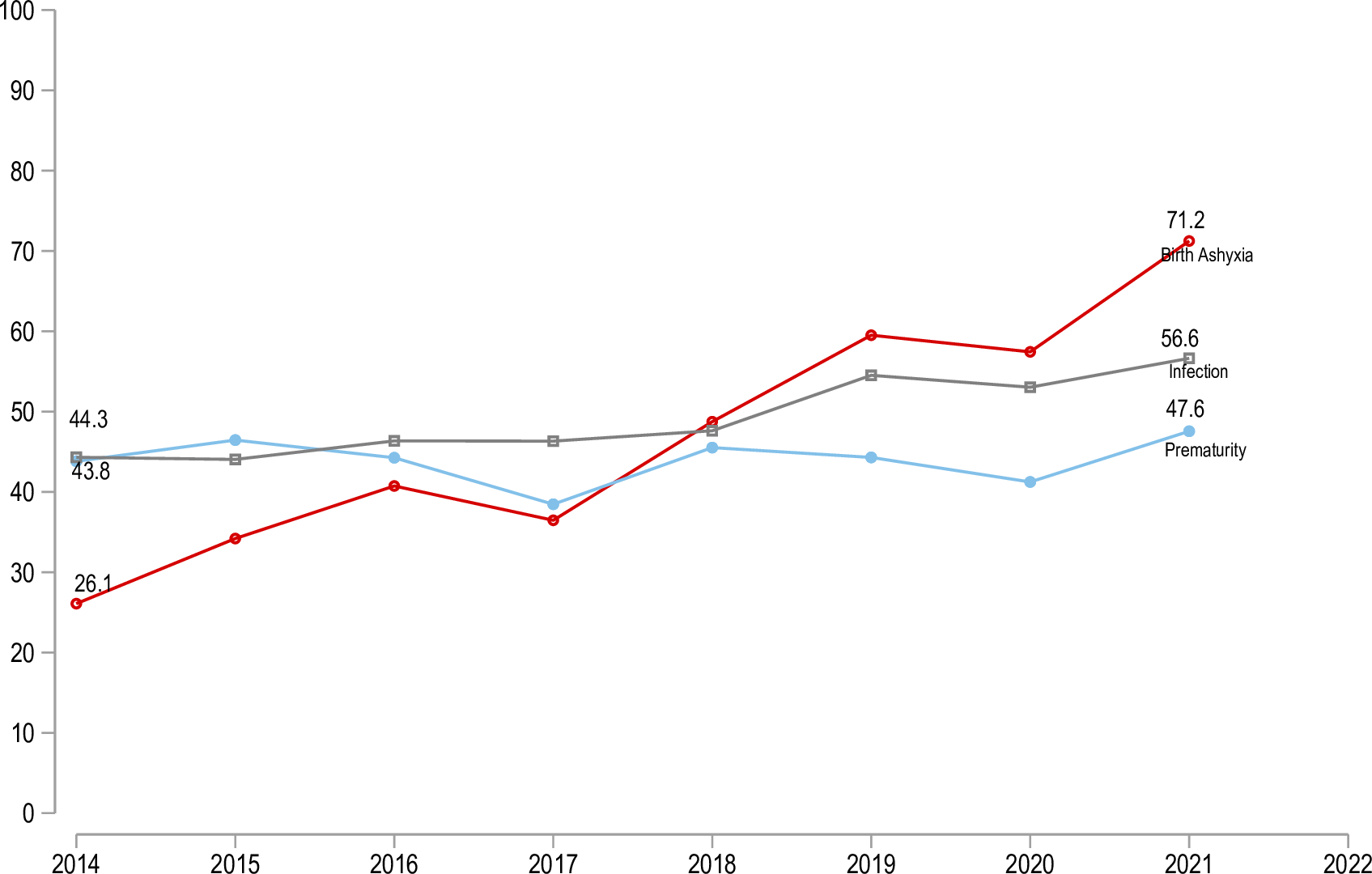

Supplement: S2 Fig — The proportion of birth asphyxia patients who were outborn increased over the period of study from 26% to 71%. This trend towards increasing admission of outborn patients was not seen in other major diagnoses. (TIF) [file pgph.0002261.s002.tif]
