# Supplementary material for: Outborn newborns drive birth asphyxia mortality rates—An 8 year analysis at a rural level two nursery in Uganda
Source: PLOS Glob Public Health. 2023 Nov 8;3(11):e0002261. doi: 10.1371/journal.pgph.0002261 (PMC10631647; doi:10.1371/journal.pgph.0002261)
Supplement: S1 Table — (DOCX) [file pgph.0002261.s003.docx]

**S1 Table: Characteristics for neonates with birth asphyxia by survival to discharge**

|  | Survived  (N = 1226)  n (%)  or median [IQR] | Died  (N = 339)  n (%)  or median [IQR] | *p* |
| --- | --- | --- | --- |
| **Maternal Demographics** |  |  |  |
| **Mother’s age (years)** (n=1374)  <20  20 - 34  ≥35 | 263 (24.4)  731 (67.9)  82 (7.6) | 67 (22.5)  211 (70.8)  20 (6.7) | 0.6 |
| **Gravida** (n=1474)  1 (primigravid)  2-3  ≥ 4 | 478 (41.1)  385 (33.1)  299 (25.7) | 134 (43.0)  109 (34.9)  69 (22.1) | 0.4 |
| **Antenatal care visits** (n=1302)  0  1-3  ≥ 4 | 3 (0.3)  444 (43.0)  586 (56.7) | 0 (0.0)  120 (44.6)  149 (55.4) | 0.6 |
| **Maternal district** (n = 1565)  Within hospital district  Outside hospital district | 453 (37.0)  773 (63.0) | 125 (36.9)  214 (63.1) | 1 |
| **Mode of transport** (mother or baby): |  |  | <0.001** |
| Ambulance | 220 (17.9) | 114 (33.6) |  |
| Another vehicle | 891 (72.7) | 205 (60.5) |  |
| Bicycle or foot | 115 (9.4) | 20 (5.9) |  |
| **At Birth** |  |  |  |
| **Birth Location** (n= 1565)  Inborn (Kiwoko)  Outborn | 700 (57.1)  526 (42.9) | 103 (30.4)  236 (69.6) | <0.001** |
| **Type of birth attendant** (n= 1565)  Doctor  Midwife/nurse  TBA, family member or other | 412 (33.6)  802 (65.4)  12 (1.0) | 99 (29.2)  233 (68.7)  7 (2.1) | 0.1 |
| **Mode of delivery** (n= 1565)  Vaginal  Caesarean section | 831 (67.8)  395 (32.2) | 252 (74.3)  87 (25.7) | 0.02* |
| **Meconium-stained fluid** (n=1039)  No  Yes | 468 (56.2)  365 (43.8) | 117 (56.8)  89 (43.2) | 0.9 |
| **Cried at birth** (n=1388)  No  Yes | 885 (81.7)  198 (18.3) | 261 (85.6)  44 (14.4) | 0.1 |
| **Apgar- 1 minute** (n=1227) | 5 (4-6) | 4 (3-5) | 0.6 |
| **Apgar- 5 minute** (n=1202) | 7 (6-8) | 5 (4-7) | 0.7 |
| **Infant characteristics** |  |  |  |
| **Sex** (n= 1565)  Female  Male | 477 (38.9)  749 (61.1) | 131 (38.6)  208 (61.4) | 0.9 |
| **Multiple birth** (n=1560)  Singleton  Multiple birth | 1167 (95.3)  57 (4.7) | 321 (95.5)  15 (4.5) | 0.9 |
| **Age at admission** (days) (n = 1565)  Day of birth  1-2  3+ days | 920 (75.0)  258 (21.0)  48 (4.0) | 237 (69.9)  90 (26.6)  12 (3.5) | 0.1 |
| **Birthweight** (n= 1546)  < 2.5kg  2.5-4.49kg  >=4.5kg | 112 (9.2)  1087 (89.6)  14 (1.1) | 50 (15.0)  282 (84.7)  1 (0.3) | 0.004* |
| **Temperature** (°C) (n=1537)  < 36.5  36.5-37.9  ≥ 38 | 710 (58.9)  394 (32.7)  102 (8.4) | 188 (56.8)  86 (26.0)  57 (17.2) | <0.001** |
| **Respiratory rate** (bpm) (n=1379)  < 30  30-60  > 60 | 17 (1.5)  502 (45.1)  593 (53.3) | 25 (9.4)  136 (50.9)  106 (39.7) | <0.001** |
| **Oxygen saturation** (%) (n = 1417)  < 90  ≥ 90 | 379 (33.9)  738 (66.1) | 177 (59.0)  123 (41.0) | <0.001** |
| **Blood sugar** (mmol/L) (n = 1441)  < 2.6  2.6-8.3  >8.3 | 69 (6.0)  879 (76.8)  196 (17.1) | 27 (9.1)  179 (60.3)  91 (30.6) | <0.001** |
| **Therapies received:** |  |  |  |
| Phototherapy (n=1545) | 249 (20.5) | 34 (10.3) | <0.001** |
| Blood transfusion (n=1555) | 14 (1.1) | 8 (2.4) | 0.09 |
| Bubble CPAP (n=1543) | 125 (10.4) | 152 (45.0) | <0.001** |

Each analysis is based on available data for that measure, as such denominators differ depending on the response. *p <0.05, **p<0.001
