# Supplementary material for: Outborn newborns drive birth asphyxia mortality rates—An 8 year analysis at a rural level two nursery in Uganda
Source: PLOS Glob Public Health. 2023 Nov 8;3(11):e0002261. doi: 10.1371/journal.pgph.0002261 (PMC10631647; doi:10.1371/journal.pgph.0002261)
Supplement: S2 Table — (DOCX) [file pgph.0002261.s004.docx]

**S2 Table. Risk factors for death among birth asphyxia patients by birth location**

|  | **All Birth Asphyxia Patients** | | **Inborn only** | **Out-born only** |
| --- | --- | --- | --- | --- |
|  | **Crude RR**  **(95% CI)** | **Adjusted RR**  **(95% CI)** | **Adjusted RR**  **(95% CI)** | **Adjusted RR**  **(95% CI)** |
| **Birth location** |  |  |  |  |
| Inborn | 1 | 1 | N/A | N/A |
| Outborn | 1.7 (1.4-2.2) ** | 2.1 (1.5-3.1) ** |  |  |
| **Mode of delivery** |  |  |  |  |
| Vaginal | 1 |  |  |  |
| Caesarean | 0.9 (0.7-1.1) |  |  |  |
| **Sex** |  |  |  |  |
| Male | 1 |  |  |  |
| Female | 1.03 (0.8-1.3) |  |  |  |
| **Birth weight** (grams) |  |  |  |  |
| <2500 | 1.3 (0.9-1.8) | 1.4 (0.8-2.3) | 0.96 (0.3-2.7) |  |
| ≥2500 | 1 | 1 | 1 |  |
| **Admission day of age** |  |  |  |  |
| Day of birth | 1 |  |  | ^ |
| 1-2 days | 1.01 (0.8-1.3) |  |  |  |
| 3+ days | 0.7 (0.4-1.3) |  |  |  |
| **Temperature** (°C) |  |  |  |  |
| <36.5 | 1.4 (1.1-1.9) * | # | 0.9 (0.4-2.1) | 1.4 (0.9-2.3) |
| 36.5-37.9 | 1 |  | 1 | 1 |
| 38+ | 1.8 (1.3-2.7) ** |  | - | 1.8 (1.2-3.0) * |
| **Respiratory rate** (bpm) |  |  |  |  |
| <30 | 3.5 (1.9-6.3) ** | 3.9 (1.9-7.8) ** | 5.0 (2.0-13.0) ** | 2.7 (1.1-6.6) * |
| 30-60 | 1 | 1 | 1 | 1 |
| >60 | 0.7 (0.5-0.9) ** | 0.6 (0.4-0.8) * | 0.5 (0.2-0.98) * | 0.6 (0.4-0.9) * |
| **Oxygen sat** (%) |  |  |  |  |
| < 90 | 2.4 (1.8-3.1) ** | 2.0 (1.4-2.7) ** | 3.6 (1.8-6.9) ** | 1.7 (1.2-2.4) * |
| ≥ 90 | 1 | 1 | 1 | 1 |
| **Blood sugar** (mmol/L) |  |  |  |  |
| <2.6 | 1.5 (0.97-2.4) | 1.4 (0.8-2.6) | 2.46 (0.97-6.2) | 1.1 (0.5-2.1) |
| 2.6-8.3 | 1 | 1 | 1 | 1 |
| >8.3 | 2.1 (1.6-2.8) ** | 1.7 (1.1-2.5) * | 0.8 (0.3-1.8) | 2.0 (1.3-3.2) * |
| **Year of admission** |  |  |  |  |
| 2014 | 1 | 1 | 1 | 1 |
| 2015 | 1.4 (0.8-2.5) | 1.8 (0.8-4.3) | 1.5 (0.5-4.6) | 2.1 (0.6-7.6) |
| 2016 | 1.6 (0.9-2.8) | 1.1 (0.5-2.9) | 0.8 (0.2-3.0) | 1.4 (0.4-5.0) |
| 2017 | 1.1 (0.6-2.0) | 1.1 (0.5-2.6) | 0.3 (0.05-1.5) | 1.8 (0.6-5.9) |
| 2018 | 1.9 (1.1-3.2) * | 2.0 (0.9-4.5) | 1.5 (0.5-4.6) | 2.0 (0.6-6.4) |
| 2019 | 1.9 (1.1-3.2) * | 2.0 (0.9-4.4) | 1.3 (0.4-4.0) | 2.4 (0.8-7.4) |
| 2020 | 1.9 (1.2-3.2) * | 2.4 (1.1-5.1) * | 1.8 (0.6-5.0) | 2.4 (0.8-7.2) |
| 2021 | 1.8 (1.1-3.0) * | 1.8 (0.8-3.9) | 2.5 (0.8-7.5) | 1.9 (0.6-5.5) |

*p<0.05, **p<0.001. ^*Given collinearity between day of admission and outborn status, day of admission was not included in multivariate (Poisson) regression of outborn patients. #Given collinearity between temperature and outborn status, temperature was not included in multivariate (Poisson) regression.*
