# Supplementary material for: Outborn newborns drive birth asphyxia mortality rates—An 8 year analysis at a rural level two nursery in Uganda
Source: PLOS Glob Public Health. 2023 Nov 8;3(11):e0002261. doi: 10.1371/journal.pgph.0002261 (PMC10631647; doi:10.1371/journal.pgph.0002261)
Supplement: Attachment — Submitted filename: Response to Reviewers.docx [file pgph.0002261.s006.docx]

PGPH-D-23-01252
Outborn Newborns Drive Birth Asphyxia Mortality Rates- an 8 Year Analysis at a Rural Level Two Nursery in Uganda
PLOS Global Public Health

Response to Reviewers

Journal Requirements:

1. Please include a complete copy of PLOS’ questionnaire on inclusivity in global research in your revised manuscript.
**This has been included with the submission.**

Please review your reference list to ensure that it is complete and correct. Any changes to the reference list should be mentioned in the rebuttal letter that accompanies your revised manuscript.
**This has been done.**

Review Comments to the Author

Reviewer #1: I would recommend adding some data about the standard criteria for the diagnosis of severe birth asphyxia (pH, BE, Apgar score, etc.).

**We agree with the importance of being clear about the diagnostic criteria, especially in a setting without advanced diagnostics. The manuscript contains this information (page 6):**

“*The diagnosis of birth asphyxia was at the discretion of the discharging physician, and utilized available information including 5-minute Apgar score <7, poor tone and/or presence of clinically suspected seizure. Although in other settings low apgars have been associated with poor outcomes[26, 27], reported apgars in this dataset are unreliable, and therefore high apgar scores are not routinely used as exclusion criteria for asphyxia by discharging physicians*.”

In addition, we added:

“*The unit did not utilize a standardized exam of patients with risk of asphyxia such a Sarnat staging or Thompson scoring*.”

Also, give more details about the COVID-19 pandemic period and what was your admission guideline during that period.

**We added (page 5):** “COVID Period- *No admission criteria changed during the COVID pandemic. The early COVID period (April through September 2020) saw 14% decrease in admissions but an increased proportion of admission with birth asphyxia (22% vs. 15%).[20]*”

You can also be more specific about the guideline used in these patients and what would be the means of improving the outcome.

No guidelines exist at Kiwoko for the care of patients with birth asphyxia, but a standardized practice does occur. We amended the text (starting at lines 125) to be more explicit about the care these patients received:

“Clinical Care of Patients with Birth Asphyxia

Throughout the period of study, care provided to patients with birth asphyxia included thermoregulation via overhead warmer, incubators and kangaroo mother care, intravenous hydration including use of IV pumps, nasogastric,cup and breast feeding, phototherapy, antibiotics, anticonvulsants, neurodevelopmental care including promoting flexed positioning, blood transfusion, basic laboratory services including complete blood count and electrolytes, CPAP therapy[24, 25] and intermittent as well as continuous pulse oximetry as needed. The unit does not have capacity for surfactant, mechanical ventilation, parenteral nutrition, central lines, blood culture, coagulation measurement, electro encephalogram, MRI or therapeutic hypothermia, and these therapies are not routinely available even via transfer to the national referral hospital located two hours travel by vehicle. Close monitoring of patients included care by nurses with an average of 9 patients to 1 nurse. Continuous physician coverage was available as needed and rounds were lead daily by a pediatrician or medical officer.”

We conclude that outcomes of neonates with birth asphyxia admitted to the Kiwoko nursery would be most impacted by improved health of babies admitted after birth elsewhere (outborns). The national guidelines proposed in the discussion including use of champions, improved staffing ratios, mentorship, intrapartum monitoring, etc may allow for both decreased asphyxia as well as improved outcomes of asphyxiated babies who require transfer to a higher level of care.

Reviewer #2: Perinatal asphyxia is responsible for a large number of NICU admissions and neonatal deaths in low- and middle-resource countries. His work is in line with other publications on the African continent.
Improvements in prenatal and perinatal care are needed to reduce the mortality of asphyxiated newborns.
This study helps to know the local reality and makes it possible to compare it with other similar African studies to adopt measures that allow expanding the information and taking action within the global health approach.

Cavallin F, Menga A, Brasili L, Maziku D, Azzimonti G, Putoto G, Trevisanuto D. Factors associated with mortality among asphyxiated newborns in a low-resource setting. J Matern Fetal Neonatal Med. 2022 Mar;35(6):1178-1183. doi: 10.1080/14767058.2020.1743670. Epub 2020 Mar 25. PMID: 32212882.

**This is a helpful study to reference and has been added as a citation.**
